# Coupling bacterial nitrogen fixation to yeast whole-cell biocatalysis enables amine production using air as the sole nitrogen source

**DOI:** 10.1186/s12934-026-03118-3

**Published:** 2026-09-16

**Authors:** Khaled Youssef, Ed W. J. van Niel, Brett Barney, Magnus Carlquist

**Affiliations:** 1 Biotechnology and Applied Microbiology, Department of Process and Life Science Engineering, Lund University, P.O. Box 124, SE-221 00 Lund, Sweden; 2 Department of Bioproducts and Biosystems Engineering, University of Minnesota, St. Paul, MN USA; 3 Biotechnology Institute, University of Minnesota, St. Paul, MN USA

**Keywords:** Biological nitrogen fixation, Whole‑cell biocatalysis, Alkylamines, Vanillylamine, *Azotobacter vinelandii*, *Saccharomyces cerevisiae*, Sequential bioprocessing, Sustainable biomanufacturing.

## Abstract

**Background:**

Alkylamine production depends on ammonium produced via the energy-intensive, fossil-based Haber-Bosch process. Diazotrophic bacteria could offer a biological alternative by supplying nitrogen directly from air to heterotrophic whole-cell biocatalysts for alkylamine production. However, their integration with yeast production systems remains largely unexplored.

**Results:**

Here, we evaluated the use of an ammonium-excreting strain of *Azotobacter vinelandii* (AZBB664) to supply nitrogen to an engineered *Saccharomyces cerevisiae* biocatalyst for vanillylamine production using air as the sole nitrogen source. A modified Burk’s medium was developed to support *S. cerevisiae* growth and enable evaluation of biologically supplied nitrogen. Co-cultivation of both organisms was feasible under diazotrophic conditions, however, vanillin oxidation by *A. vinelandii* prevented effective vanillylamine production. To address this, a sequential process was implemented in which *A. vinelandii* was first used to generate ammonium in a modified Burk’s medium, followed by cell removal and use of the resulting medium (AZM1) for yeast cultivation. In this medium, the yeast strain CEN.PK 113-7d exhibited a growth rate of 0.27 h^− 1^ and reached higher final cell density compared to the control medium supplemented with synthetic ammonium. Cultivation in AZM1 was associated with a more stable pH profile, which may have contributed to the improved growth of the yeast. *S. cerevisiae* strain TMBNM033 expressing recombinant transaminase and alanine dehydrogenase was able to convert vanillin to vanillylamine, although a significant amount of vanillic alcohol was also observed due to competing endogenous reductase activity.

**Conclusions:**

*A. vinelandii-*derived medium enables yeast growth and whole-cell reductive amination using air as the sole nitrogen source. This demonstrates that biologically fixed nitrogen can directly sustain heterotrophic whole-cell biotransformations, establishing a strategy for coupling diazotrophy with biocatalytic production systems.

**Graphical Abstract:**

**Figure:**
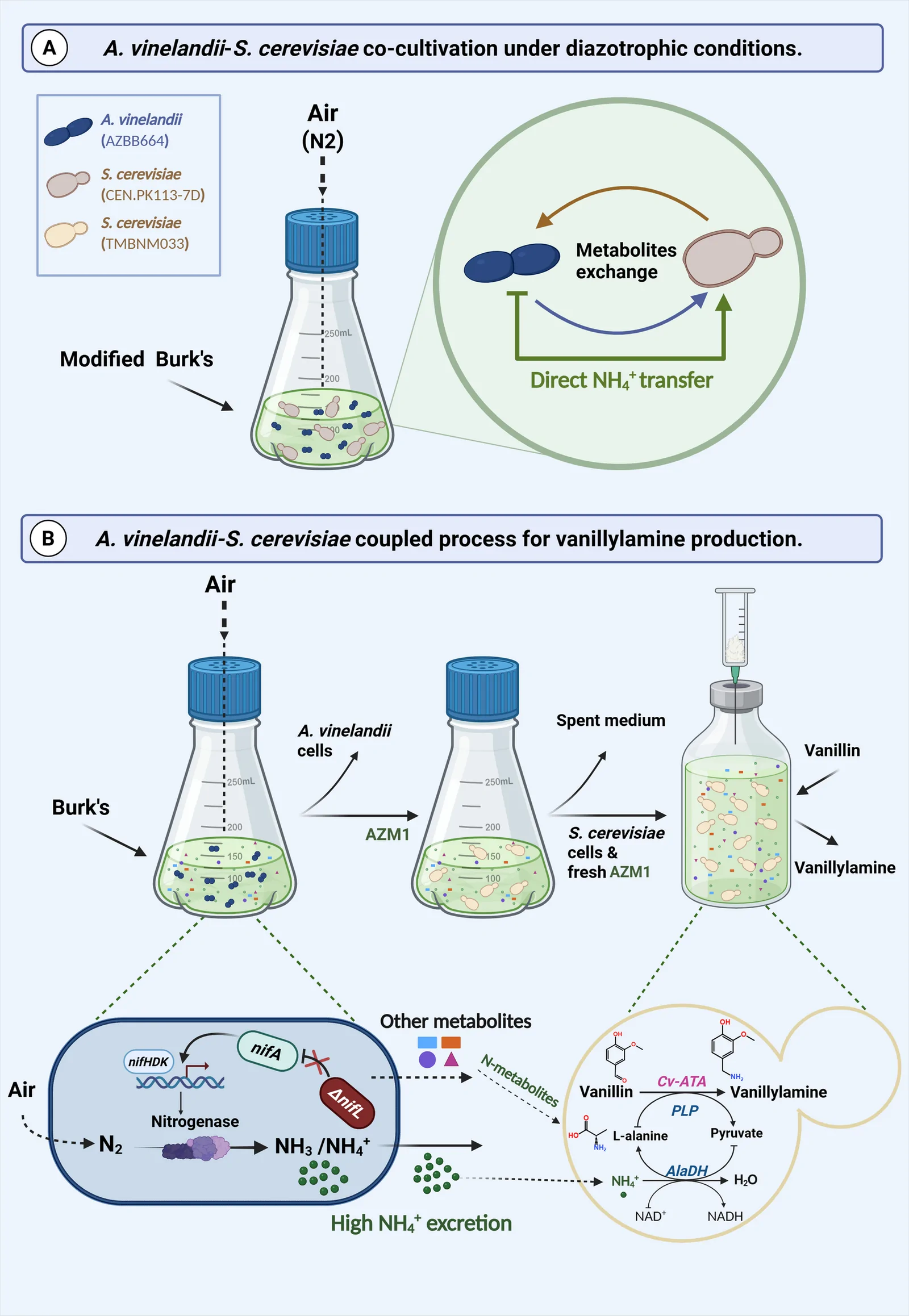

**Supplementary Information:**

The online version contains supplementary material available at https://doi.org/10.1186/s12934-026-03118-3.

## Background

Amines are widely used as building blocks in polymers, agrochemicals, and pharmaceuticals [1]. Biocatalysis provides an attractive alternative to conventional chemical synthesis, but current processes rely on externally supplied reduced nitrogen, typically in the form of inorganic ammonium derived from the Haber-Bosch process [2]. Operating with metal catalysts under extreme conditions (> 400 °C, 150–200 bar), this process demands high energy inputs (> 30 GJ/ tonne NH_3_) largely met by fossil fuels [3–5]. Consequently, it accounts for approximately 1.2% of globa CO_2_ emissions, and 2% of global energy consumption [4, 6]. Currently, most whole-cell biocatalytic routes to amines are based on recombinant *Escherichia coli* [2], or yeast [7] cultivated in media supplemented with excess ammonium or specific amine donors, ultimately limiting overall process sustainability and economic viability.

*Saccharomyces cerevisiae* has emerged as a versatile whole-cell biocatalyst for amine production across a range of applications [8], including lignin-derived compounds such as vanillylamine [7, 9]. In these systems, nitrogen incorporation typically relies on externally supplied ammonium and amino acids, which function both as nitrogen sources for cellular metabolism and as direct amine donors for the recombinant transaminases.

Biological nitrogen fixation offers a potential route to generate reduced nitrogen from atmospheric N₂ under mild physiological conditions. This biologically generated fixed nitrogen could, in principle, be used to support the growth and catalytic activity of a yeast whole-cell biocatalyst without the addition of synthetic ammonium. The diazotrophic soil bacterium *Azotobacter vinelandii* converts N_2_ to ammonium aerobically under ambient temperature and pressure conditions employing metal-dependent nitrogenase complex enzyme [3, 10]. Because this reaction is energetically demanding, requiring at least 16 ATP molecules and 8 electrons for each N_2_ molecule fixed [10], expression of the nitrogen-fixation machinery is tightly controlled by the NifL-NifA regulatory system [10, 11]. NifA activates transcription of nitrogen-fixation (*nif*) genes whereas NifL functions as an anti-activator that suppresses NifA in response to nitrogen availability, and changes in cellular redox and energy status [12]. Disruption of *nifL* results in partial deregulation of the *nif* genes from these signals, permitting sustained nitrogenase activity and extracellular ammonium accumulation [13]. In a deregulated *A. vinelandii* strain, an extracellular NH_4_^+^ concentration of around 21.5 mM has been reported to a net yield approximately 1 mol NH_4_^+^ per 1.35 mol sucrose consumed [14]. Furthermore, *A. vinelandii* has previously been found to support co-cultivation of heterotrophic production hosts, including *Yarrowia lipolytica* and *E. coli* by generating nitrogen in situ rather than supplied externally [15, 16]. However, several factors may limit such an approach, including differences in physiology, competition for substrates, and uncertainty regarding whether extracellular ammonium can sustain downstream biocatalytic reactions. It remains unresolved whether nitrogen fixed by *A. vinelandii* can be transferred to *S. cerevisiae* at sufficient levels to sustain both growth and whole-cell biocatalytic activity.

In this study, ammonium production by *A. vinelandii* under diazotrophic conditions was first evaluated in relation to the nitrogen requirements of *S. cerevisiae*. Co-cultivation was then explored to assess physiological compatibility and substrate competition. Finally, the feasibility of directly coupling biologically fixed nitrogen to vanillylamine production, as a representative whole-cell amination reaction, was assessed.

## Materials and methods

### Strains and media

All strains used in this study are listed in Table 1. Strains were maintained in 25% (v/v) glycerol stocks at -80 °C and routinely recovered by streaking on the appropriate agar medium prior cultivation. *S. cerevisiae* strains were maintained on yeast peptone dextrose (YPD) medium containing: 10 g/L yeast extract, 20 g/L peptone, 20 g/L glucose, and 15 g/L agar. *A. vinelandii* strains were maintained on Burk’s medium containing: 0.8 g/L K_2_HPO_4_, 0.2 g/L KH_2_PO_4_, 0.016 CaSO_4_, 0.41 g/L MgSO_4_, 20 g/L sucrose, 0.012 g/L FeCl_3_.6H_2_O, 0.003 g/L Na_2_MoO_4_.2H_2_O, and 15 g/L agar [17]. For non-diazotrophic growth experiments, the medium was supplemented with 1 g/L (18.78 mM) NH_4_Cl. For yeast monoculture and cocultivation experiments, a modified Burk’s medium supplemented with 1% (v/v) trace element solution and 0.1% (v/v) vitamin solution was used. Both trace elements and vitamins stocks were prepared according to the Delft medium formulation [18]. The trace element solution was prepared as a 100× stock and contained, 3 g/L FeSO_4_·7H_2_O, 4.5 g/L ZnSO_4_·7H_2_O, 4.5 g/L CaCl_2_·6H_2_O, 0.84 g/L MnCl_2_·2H_2_O, 0.3 g/L CoCl_2_·6H_2_O, 0.3 g/L CuSO_4_·5H_2_O, 0.4 g/L Na_2_MoO_4_·2H_2_O, 1 g/L H_3_BO_3_, 0.1 g KI, and 15 g/L Na_2_EDTA. The vitamin solution was prepared as a 1000x stock and contained 0.05 g/L biotin dissolved in 10 mL 0.1 M of NaOH, 1 g/L Ca-pantothenate, 1 g/L nicotinic acid, 25 g myo-inositol, 1 g/L thiamine hydrochloride, 1 g/L pyridoxal hydrochloride, and 0.2 g/L *p*-aminobenzoic acid.

**Table 1:** *Azotobacter vinelandii* and *Saccharomyces cerevisiae* strains used in this study

Organism	Strains	Genotypic treats	Reference
*Azotobacter vinelandii*	ATCC BAA-1303 (DJ)	Wild type	[19] ATCC collection.
	AZBB664	Intergenic region (*Avin_11360-Avin_11370*)::kan^R^-*nifA*-segment; Δ*nifA2;* Δ*nifLA*	[20]
*Saccharomyces cerevisiae*	CEN.PK113-7D	Wild type	[21] EUROSCARF collection
	TMBNM033	CEN.PK 2–1 C; *TDH3*p-*CvTA6x-ADH1*t; *TEF1*p-*BsAlaDH*-*ADH1*t; Δ*ADH6:AurR*	[9]

### Cultivation of *S. cerevisiae* and *A. vinelandii* in modified Burk’s media

Precultures of *S. cerevisiae* strains were initiated from single colonies grown on YPD agar and cultivated overnight in 25 mL modified Burk’s medium supplemented with 1 g/L NH_4_Cl and 10 g/L sucrose or glucose at 30 °C and 180 rpm. Cells were harvested by centrifugation, washed three times with sterile Burk’s salt solution, and resuspended in fresh medium. Experimental cultures were inoculated to an initial OD_600_ of 0.3 in 250 mL baffled shake flasks containing 25 mL modified Burk’s medium (pH 7.3) supplemented with the indicated NH_4_Cl concentrations and incubated at 30 °C and 180 rpm (Innova 43, Eppendorf) for 48 h.

Precultures of *A. vinelandii* ATCC BAA-1303 and AZBB664 were initiated from colonies on agar plates and cultivated in 25 mL Burk’s medium in 250 mL baffled shake flasks for 24–26 h at 30 °C and 180 rpm. Experimental cultures were inoculated to an initial OD_600_ of 0.3 in 250 mL baffled shake flasks containing 30 mL Burk’s medium and incubated under the same conditions.

### Co-cultivation of *A. vinelandii* and *S. cerevisiae* and analysis of community composition

Co-cultivation experiments were performed to assess the compatibility of *A. vinelandii* and *S. cerevisiae* under diazotrophic and ammonium-supplemented conditions. Co-cultures were established in 30 mL of modified Burk’s medium in 250 mL baffled shake flasks and inoculated with *A. vinelandii* AZBB664 and *S. cerevisiae* CEN.PK113-7D to initial OD_600_ values of 0.3 and 0.5, respectively.

Two cultivation regimes were evaluated: diazotrophic co-cultivation without addition of fixed nitrogen, and co-cultivation supplemented with NH_4_Cl (1 g/L, 18.78 mM). Cultivations were carried out in baffled shake flasks under the incubation conditions described above.

Samples were collected at defined time points during cultivation. Community dynamics were analysed by flow cytometry to resolve bacterial and yeast subpopulations and quantify viable cells within each population, expressed as active fluorescent units (AFU/mL). In parallel, colony-forming units (CFU/mL) were determined by plating serial dilutions on selective agar media to validate population trends.

At each sampling point, culture supernatants were collected after centrifugation for extracellular metabolite analysis. Culture pH was measured at 0, 12 and 48 h of cultivation.

### Vanillin utilization by *A. vinelandii*

Vanillin utilization by *A. vinelandii* AZBB664 was assessed in monoculture to determine whether the diazotrophic strain could compete for the aromatic substrate used in the yeast biocatalytic phase. Precultures were prepared as described above, and experimental cultures were established in Burk’s medium supplemented with 5 mM vanillin. Cultures were inoculated to an initial OD_600_ of 0.3 in baffled shake flasks and incubated at 30 °C and 180 rpm under the same conditions as diazotrophic monocultures. Culture supernatants were collected by centrifugation (4,000 × g, 10 min, 20 °C) for HPLC analysis of vanillin, vanillic acid, and vanillyl alcohol.

### Whole-cell bioconversion using *A. vinelandii-*derived medium (AZM1)

*A. vinelandii* AZBB664 was cultivated under diazotrophic conditions to generate a nitrogen-containing, cell-free medium (AZM1). Cultures were inoculated to an initial OD_600_ of 0.3 in 1 L baffled shake flasks containing 120 mL Burk’s medium without fixed nitrogen and incubated for 24 h at 30 °C and 180 rpm. Cells were removed by centrifugation, and the supernatant was sterile-filtered (0.22 μm), adjusted to pH 6.5 ± 0.1 using 5 M H_2_SO_4_, and supplemented with 20 g/L sucrose, 1% (v/v) trace element solution, and 0.1% (v/v) vitamin solution. The resulting medium (AZM1) was used directly for subsequent yeast cultivation and biocatalysis. The protein content in the AZM1 medium was determined with the Bradford method [22].

The *S. cerevisiae* production strain TMBNM033 was cultivated in AZM1 medium for biomass generation. Precultures were initiated from single colonies in 5 mL AZM1, and experimental cultures were inoculated to an initial OD_600_ of 0.5 and incubated for 24 h at 30 °C and 180 rpm. Cells were harvested by centrifugation (3,500 × g, 10 min, 20 °C) and resuspended in fresh reaction medium to an OD_600_ of 5.0 ± 0.4.

Bioconversion reactions were performed in either AZM1 or modified Burk’s medium supplemented with NH_4_Cl (1 g/L, 18.78 mM) as a fixed-nitrogen control. Vanillin (5 mM) and ethanol (10 g/L) were added as substrate and co-substrate, respectively. Reactions were carried out in 60 mL sealed serum vials containing 30 mL medium and incubated at 30 °C and 180 rpm for 48 h. To evaluate different oxygen regimes, reactions were carried out under both oxygen-limited and anaerobic conditions. Anaerobiosis was achieved by sparging the vials with nitrogen gas prior to incubation for 1 h, whereas oxygen-limited conditions were maintained without sparging as described by [9]. Samples were collected at defined time points for analysis of substrate consumption and product formation.

### Growth monitoring and flow cytometry quantification of the coculture composition

Cell growth was monitored by measuring optical density at 600 nm (OD_600_) using an Ultrospec 2100pro spectrophotometer (Amersham Biosciences, Sweden).

Co-culture growth dynamics and viability of *S. cerevisiae* and *A. vinelandii* were analysed by flow cytometry using an Accuri C6 flow cytometer (BD Biosciences, USA) equipped with a 488 nm laser. Cell suspensions were diluted in 500 µl phosphate-buffered saline (PBS, pH 7.3) to an OD_600_ of 0.1–0.3 and stained with SYBR Green I and propidium iodide (PI) (final concentration 10 µg/mL). Samples were incubated for 15 min at room temperature in the dark prior to analysis. Data acquisition was performed at medium flow rate with an FSC-H threshold of 80,000, and 10,000 events were recorded per sample. Initial gating based on forward and side scatter (FSC-H and SSC-H) was applied to remove background noise and define the total cell population. A second FSC/SSC-based gate was used to discriminate between *S. cerevisiae* and *A. vinelandii* populations. Within each population, fluorescence-based gating was applied using SYBR Green I (FL1-H, 530/30 nm) and propidium iodide (FL3-H, ≥ 670 nm) to distinguish intact and permeabilised cells. Active fluorescent units (AFU/mL) were defined as the concentration of intact cells (SYBR Green I-positive, PI-negative) per millilitre and were determined separately for *S. cerevisiae* and *A. vinelandii*. Unstained samples were used to define background fluorescence. Untreated cells served as live controls, while cells treated with 70% (v/v) ethanol for 30 min were used as dead controls. These controls were used to establish and validate gating strategies. Data were analysed using FlowJo™ software (v10.8.1, USA).

Viable cell counts were determined by colony-forming unit (CFU) assays. Co-culture samples were serially diluted in sterile PBS and plated (100 µl) onto selective agar media. Burk’s agar was used for *A. vinelandii* and YPD agar for *S. cerevisiae*. Plates were incubated at 30 °C, and colonies were counted after 2 days for *S. cerevisiae* and 4 days for *A. vinelandii*.

### Quantification of extracellular ammonium

Extracellular ammonium concentrations were determined using a colorimetric assay based on o-phthalaldehyde (OPA) (Fluoraldehyde™ o-Phthaldialdehyde Reagent Solution, Thermo Scientific, United States). Calibration curves were prepared in Burk’s medium using NH_4_Cl standards (0–20 mM). Samples and standards (20 µl) were transferred to a 96-well microplate, mixed with 200 µl OPA reagent, and incubated in the dark for 30 min at room temperature. Absorbance was measured at 405 nm using a Multiskan FC microplate photometer (Thermo Scientific, United States), and ammonium concentrations were calculated from the standard curve. The limit of detection (LOD) and limit of quantification (LOQ) were determined from 10 independent measurements of blank Burk’s medium using LOD = 3.3 (σ/S) and LOQ = 10 (σ/S), where σ is the standard deviation of the blank signal and S is the slope of the calibration curve. The LOD and LOQ were 74 µM and 225 µM, respectively.

### High-performance liquid chromatography (HPLC) analysis of extracellular metabolites

Sugars and fermentation by-products (sucrose, glucose, fructose, ethanol, glycerol, and acetate) were analysed by HPLC using a Waters system equipped with an Aminex HPX-87 H ion-exclusion column (7.8 × 300 mm; Bio-Rad, USA) and a refractive index detector (Waters 2414, USA). The mobile phase consisted of 5 mM H_2_SO_4_ at a flow rate of 0.6 mL min^− 1^, and the column temperature was maintained at 60 °C.

Vanillin consumption and formation of vanillylamine, vanillic acid, and vanillyl alcohol were analysed by HPLC using a Waters system equipped with a C18 column (4.6 × 150 mm). Reverse-phase chromatography was performed at room temperature under isocratic conditions (65% solvent A, 35% solvent B) at 1 mL min^− 1^. Solvent A consisted of water with 0.1% (v/v) trifluoroacetic acid, and solvent B was acetonitrile. Detection was performed at 230 nm and 281 nm with an UV detector (UV/Vis detector 2489, Milford, USA).

### Calculations and statistical analysis

All experiments were performed in biological triplicates unless otherwise stated.

Biotransformation yield (mol mol^− 1^) of vanillylamine was calculated as the molar concentration of vanillylamine produced after 48 h divided by the net molar consumption of vanillin over the same period.

Specific growth rates (µ) of *S. cerevisiae* (CEN.PK113-7D) were determined during the exponential phase by linear regression of ln(OD_600_) versus time. Monod kinetic parameters, including the maximum specific growth rate (µ_max_) and the half-saturation constant (*K*_*s*_), were estimated by nonlinear least-squares regression using R (v4.3.3). Model adequacy was evaluated based on residual distribution, coefficient of determination (R²), and residual standard error (RSE). Differences between substrate concentrations were assessed by one-way analysis of variance (ANOVA), followed by Tukey’s honestly significant difference (HSD) test (*p* < 0.05).

Growth dynamics of *A. vinelandii* strains (ATCC BAA-1303 and AZBB664) were analysed using GraphPad Prism 11. A two-way mixed-effects model was applied, with strain and time as fixed effects and biological replicate as a random effect to account for repeated measurements. Model assumptions were evaluated by inspection of residual normality and homogeneity of variance. When significant interaction effects were observed, post hoc comparisons between strains at individual time points were performed using the Holm-Šidák correction for multiple comparisons.

## Results

### Evaluation of modified Burk’s medium for cultivation of *S. cerevisiae*

Burk’s medium supports growth of *A. vinelandii* but differs substantially from Delft mineral medium commonly used for *S. cerevisiae*, most notably in the absence of ammonium and the presence of elevated iron and molybdenum concentrations (Supplementary Table S1).

To establish a defined medium suitable for subsequent co-cultivation experiments, Burk’s medium was supplemented with trace elements and vitamins to support *S. cerevisiae* physiology. The original concentrations of iron and molybdenum were retained to ensure compatibility with *A. vinelandii* nitrogen fixation via the nitrogenase enzyme complex.

As an initial step, the ability of *S. cerevisiae* CEN.PK113-7D to grow in the modified Burk’s medium was evaluated under aerobic batch conditions with excess ammonium. Growth was comparable to Delft mineral medium, with similar specific growth rates (µ = 0.14 ± 0.01 vs. 0.15 ± 0.03 h⁻¹) and final optical densities (OD_600_ = 6.05 ± 0.07 vs. 6.1 ± 0.14) (Supplementary Fig. S1), demonstrating that the medium supports robust yeast growth.

**Fig. 1:**
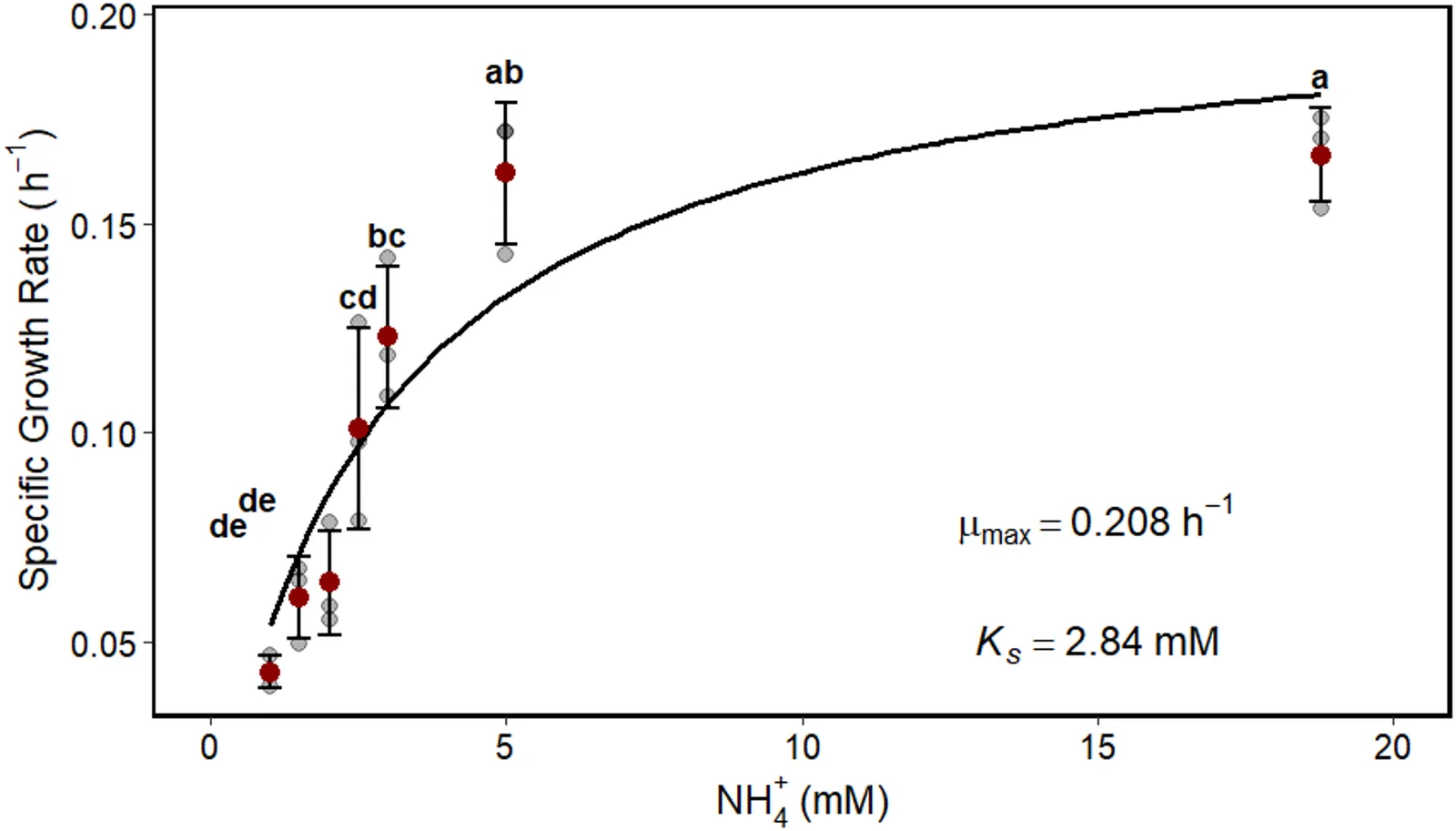
Kinetic characterization and statistical validation of *S. cerevisiae* growth under different initial ammonium concentrations. The solid line represents the non-linear regression fit to the Monod model. Red dots indicate mean specific growth rates (µ) with standard deviation(*N* = 3) while light grey dots represent individual biological replicates. Different letters **a**–**e** denote statistically significant differences (Tukey’s HSD. *p* < 0.05)

To quantify the relationship between ammonium availability and yeast growth in this medium, batch cultivations were performed with initial ammonium concentrations ranging from 0 to 18.8 mM. Specific growth rates followed Monod-type kinetics, and fitting to the Monod model yielded an apparent maximum specific growth rate (µ_max_) of 0.208 ± 0.019 h⁻¹ and a half-saturation constant (*K*_*s*_) of 2.84 ± 0.62 mM (Fig. 1). The model provided an excellent fit to the experimental data with a residual standard error (RSE) = 0.022. Ammonium concentration had a significant effect on growth (one-way ANOVA, F_6, 14_ = 33.67, *p* < 0.001). Post hoc Tukey HSD analysis showed that growth rates at 5 mM and 18.8 mM ammonium were not significantly different (µ = 0.180 vs. 0.186 h⁻¹, *p* = 0.99), indicating that near-maximal growth is achieved at approximately 5 mM (Fig. 1; Supplementary Table S2). In contrast, growth was strongly limited at concentrations below 2 mM, defining a threshold for nitrogen-limited growth.

### Growth kinetics and ammonium production profiles of *A. vinelandii* strains

**Fig. 2:**
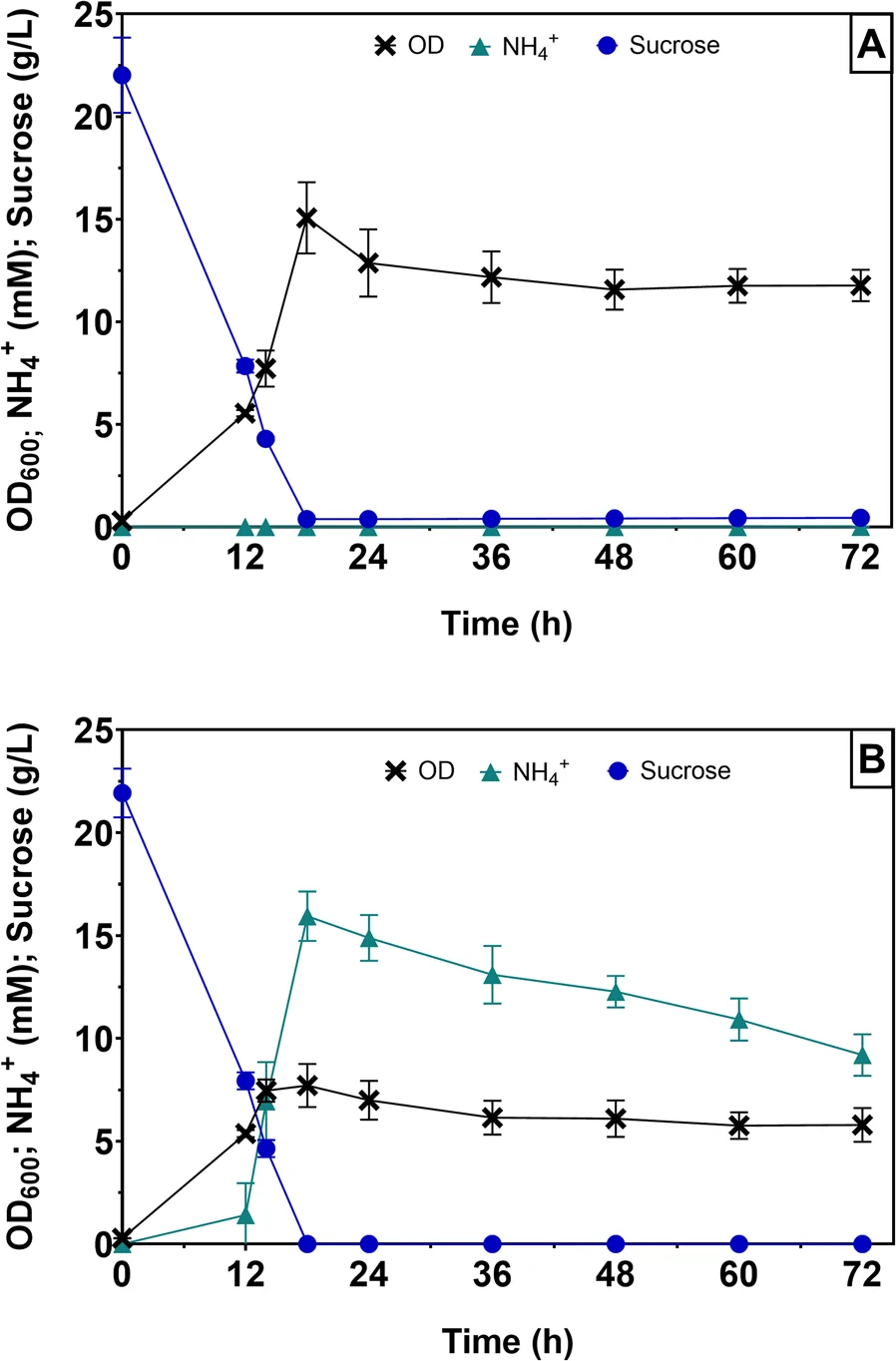
Growth kinetics, sucrose consumption, and extracellular ammonium production profiles of *A. vinelandii* strains. **A** Wild-type ATCC BAA-1303; **B** Deregulated strain AZBB664. Data points indicate averages and error bars represent standard deviation (A: *N* = 3; B: *N* = 5)

To evaluate the potential of *A. vinelandii* as a biological nitrogen donor, strains ATCC BAA-1303 (DJ) and AZBB664 were characterized in monoculture over 72 h in Burk’s sucrose medium by monitoring biomass formation, residual sucrose, and extracellular ammonium concentrations (Fig. 2). Strain ATCC BAA-1303 (DJ) was used as the reference strain, whereas AZBB664 is a previously engineered *ΔnifL* mutant reported to excrete elevated levels of ammonium [20].

Both strains rapidly consumed sucrose, with near-complete depletion by 18 h, indicating similar patterns of carbon utilization. Despite this, clear differences in biomass formation were observed, with comparable growth profiles up to 14 h, after which significant differences became apparent at 18 h (Supplementary Table S3). The final maximum OD_600_ for ATCC BAA-1303 was 2-fold higher than for AZBB664 (15.1 ± 1.7 compared to 7.7 ± 1.0 at 18 h).

In cultures of ATCC BAA-1303, ammonium remained below the detection limit throughout the experiment. In contrast, AZBB664 accumulated extracellular ammonium to 15.95 ± 1.20 mM at 18 h, followed by a gradual decline. The transient accumulation indicates net ammonium excretion during the active growth phase. Based on its ability to accumulate extracellular ammonium, AZBB664 was selected as the nitrogen-supplying strain for subsequent co-cultivation experiments with *S. cerevisiae*.

### Population dynamics and extracellular metabolite profiles in *A. vinelandii* - *S. cerevisiae* co-cultures

**Fig. 3:**
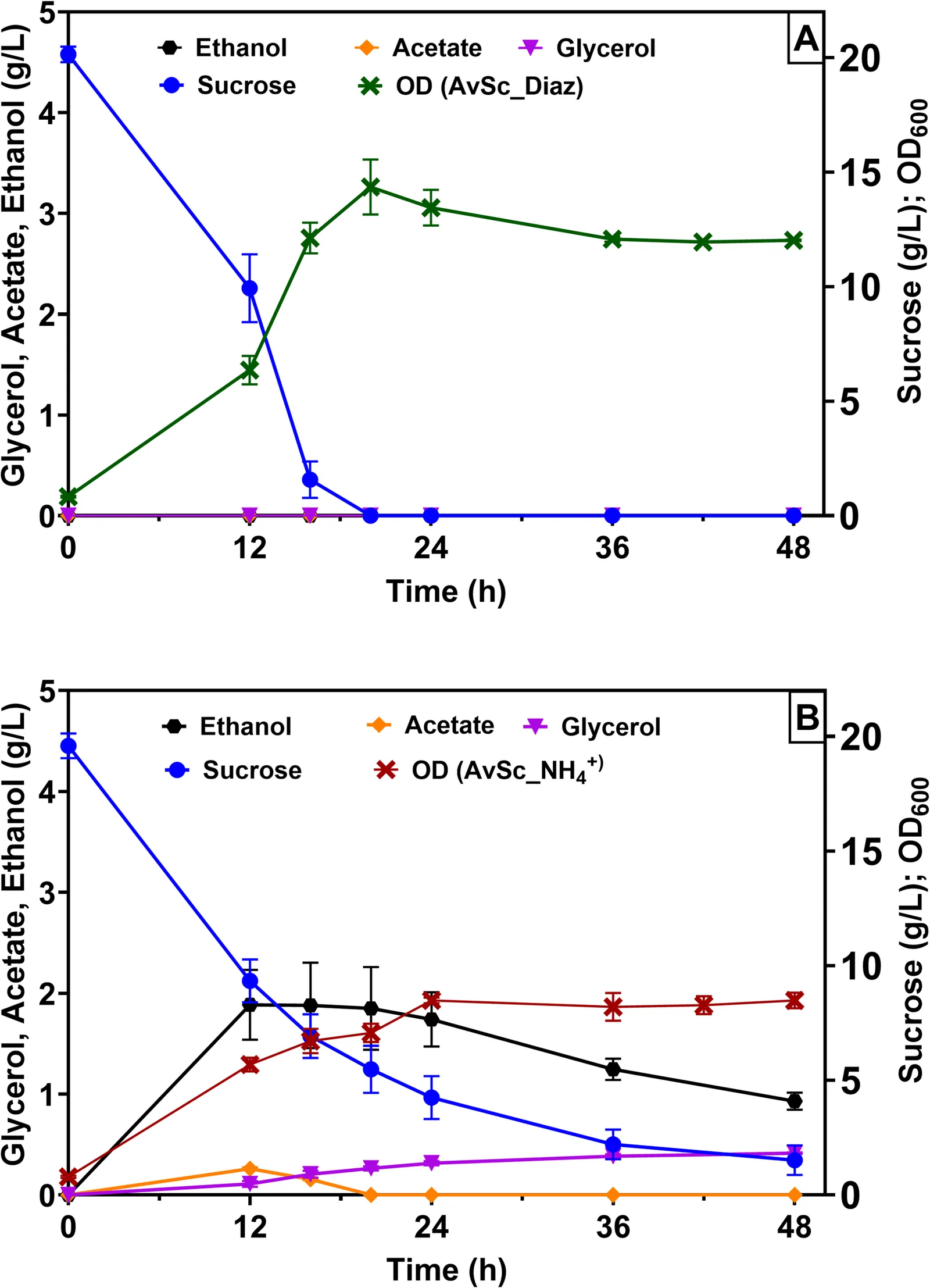
Growth and extracellular metabolite profiles of *A. vinelandii* (AZBB664) and *S. cerevisiae* (CEN.PK113-7D) during simultaneous cocultivation. Growth profiles and extracellular metabolite in modified Burk’s medium under **A** diazotrophic and **B** ammonium supplementation conditions. Data points indicate averages and error bars represent standard deviation (*N* = 3)

Co-cultivation of *A. vinelandii* AZBB664 and *S. cerevisiae* CEN.PK113-7D was evaluated in modified Burk’s medium under diazotrophic conditions and in the presence of supplemented ammonium (Fig. 3). Under ammonium-supplemented conditions, the co-culture reached an OD_600_ of 5.69 ± 0.30 at 12 h, after which growth slowed, and the culture entered stationary phase at approximately 8.48 ± 0.13 by 24 h. Under diazotrophic conditions, the initial growth was comparable (OD_600_ 6.26 ± 0.62 at 12 h) but continued beyond this point to reach a higher maximum OD_600_ of 14.35 ± 1.20 at 20 h, followed by a slight decline to 12.03 ± 0.04.

Sucrose was rapidly consumed under both conditions and was nearly depleted after 20 h. Under diazotrophic conditions, ethanol, glycerol, and acetate remained at low concentrations throughout the cultivation. In contrast, ammonium-supplemented cultures accumulated ethanol to 1.88 g/L, together with lower levels of glycerol and acetate (Fig. 3A, B). The accumulation of ethanol is consistent with the respiro-fermentative metabolism of *S. cerevisiae* under aerobic batch conditions. Notably, ethanol was not consumed during cultivation, indicating limited utilisation by *A. vinelandii* under these conditions. Acetate, on the other hand, was reassimilated indicating *A. vinelandii* activity [23].

**Fig. 4:**
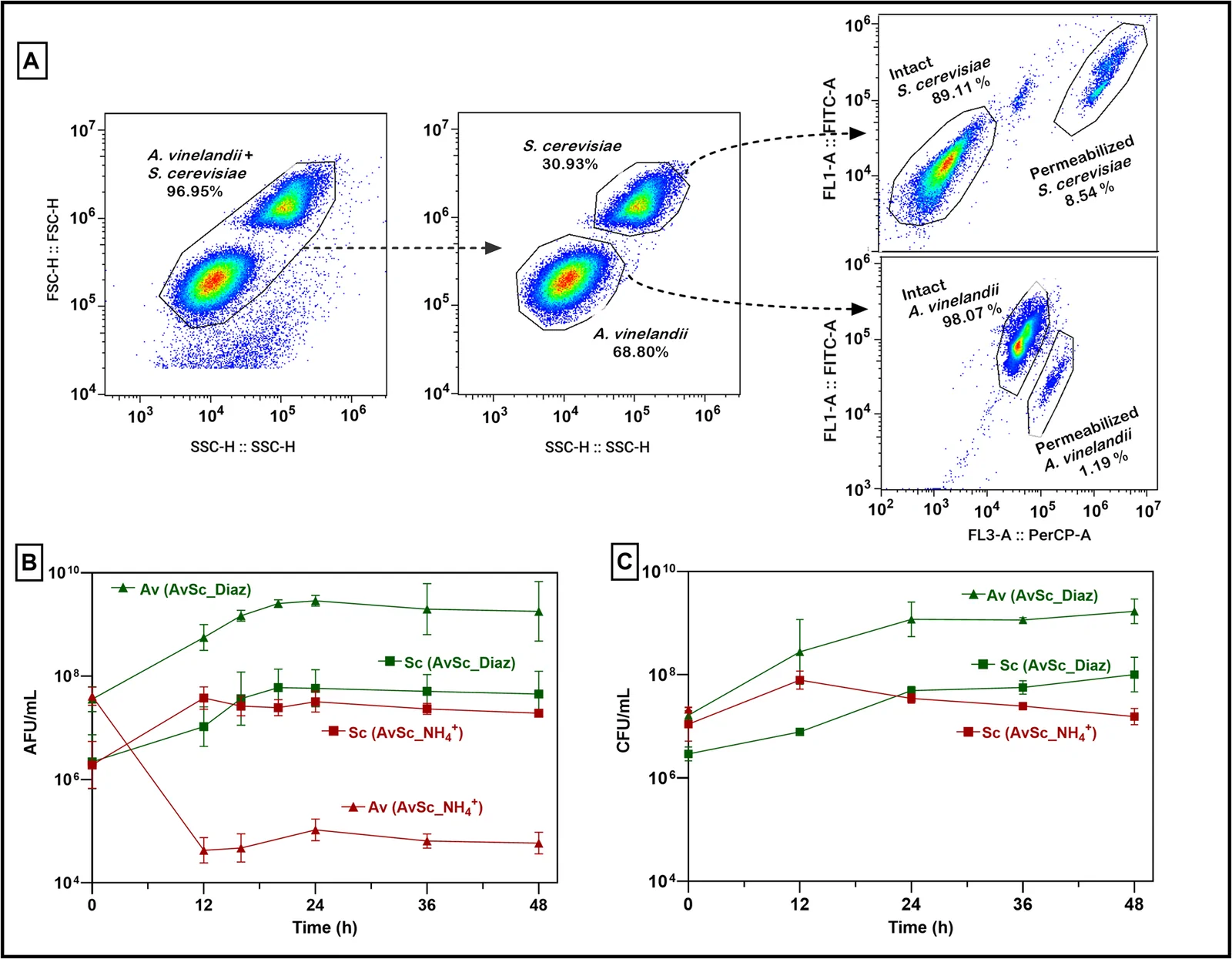
Population dynamics viability of *A. vinelandii* (AZBB664) and *S. cerevisiae* (CEN.PK113-7D) during simultaneous cocultivation under diazotrophic and ammonium-supplemented conditions. **A** Representative flow cytometry gating strategy used to resolve *A. vinelandii* and *S. cerevisiae* subpopulations in co-culture and distinguish intact (live) from permeabilized (dead) cells using SYBR Green I and propidium iodide (PI) staining. **B** Population dynamics determined by flow cytometry and expressed as active fluorescent units (AFU/mL). **C** Population dynamics of the same cocultures determined by agar plating and expressed as colony-forming units (CFU / mL). *A. vinelandii* counts fell below the limit of detection in ammonium-supplemented co-cultures. Symbols: (AvSc_Diaz) refers to cocultivation under diazotrophic conditions; (AvSc_NH_4_^+^) refers to cocultivation under ammonium supplementation conditions. Data points indicate averages and error bars represent standard deviation (*N* = 3)

Population dynamics were analysed by flow cytometry in combination with CFU measurements. The two organisms were resolved based on differences in forward and side scatter signals and staining with SYBR Green and PI enabled discrimination between intact and permeabilised cells, allowing estimation of population sizes for both species (Fig. 4A).

Under diazotrophic conditions, both *A. vinelandii* and *S. cerevisiae* increased in abundance, reaching 2.57 × 10⁹ AFU/mL and 7.67 × 10⁷ AFU/mL at 20 h, respectively. Cell numbers remained stable thereafter, indicating entry into stationary phase. Under these conditions, the pH remained stable or increased slightly towards alkaline values at the end of the cultivation. In contrast, under ammonium-supplemented conditions, *S. cerevisiae* increased to 4.11 × 10⁷ AFU/mL, while *A. vinelandii* declined rapidly to 4.73 × 10⁴ AFU/mL within the first 12 h. The loss of the bacterial population was observed in both AFU and CFU measurements and coincided with rapid acidification of the medium (Fig. 4B, C).

### Whole-cell bioconversion of vanillin to vanillylamine

**Fig. 5:**
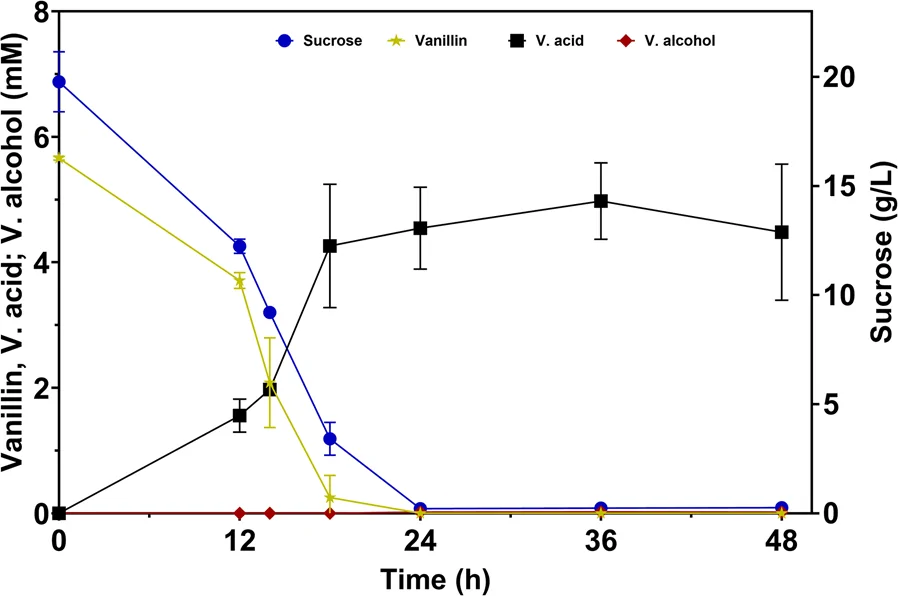
Conversion of vanillin by *A. vinelandii* AZBB664 in monoculture. Time-course profile of vanillin consumption, product formation, and growth of *A. vinelandii* AZBB664 cultivated in Burk’s medium supplemented with vanillin. Data points indicate averages and error bars represent standard deviation (*N* = 2)

The ability of *A. vinelandii* AZBB664 to support growth of *S. cerevisiae* under diazotrophic conditions prompted evaluation of its compatibility with bioconversion of vanillin to vanillylamine. However, *A. vinelandii* rapidly oxidised vanillin to vanillic acid, indicating high endogenous vanillin dehydrogenase activity (Fig. 5). This rapid conversion suggested that vanillin would be efficiently consumed by *A. vinelandii* in co-culture, thereby outcompeting yeast-catalysed amination and resulting in low vanillylamine yields.

To avoid this substrate competition, a temporally separated process was established, in which ammonium production by *A. vinelandii* was decoupled from yeast growth and bioconversion. *A. vinelandii* AZBB664 was first cultivated under diazotrophic conditions, resulting in extracellular accumulation of 15.8 ± 0.7 mM ammonium and 25.14 ± 6.39 µg/mL protein. Removal of bacterial cells yielded a cell-free, nitrogen-containing medium (AZM1), which was subsequently used to support growth of *S. cerevisiae*.

**Fig. 6:**
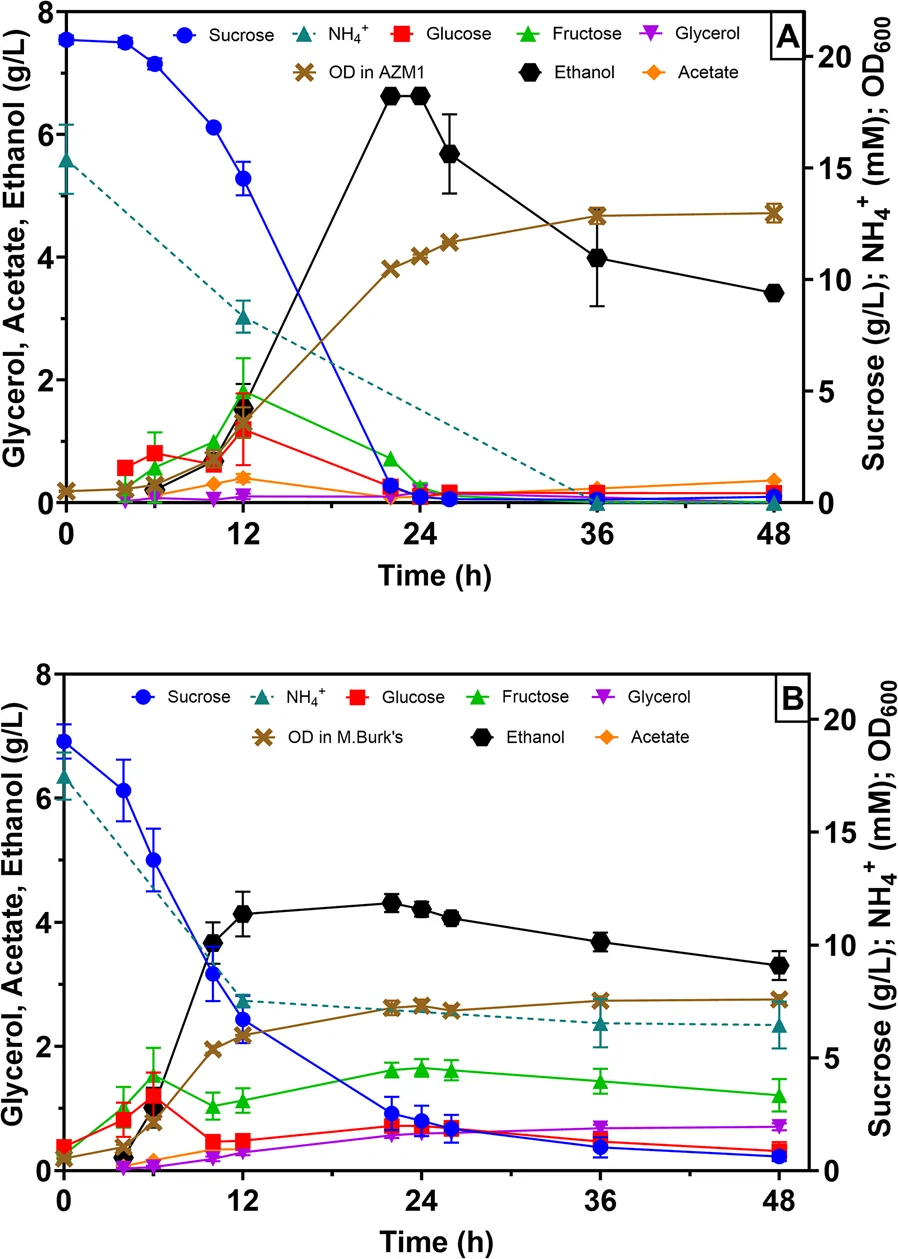
Growth and extracellular metabolite profiles of *S. cerevisiae* CEN.PK113-7D strain in **A** AZM1 and **B** ammonium-supplemented modified Burk’s medium. Data points indicate averages and error bars represent standard deviation (*N* = 3)

Growth in AZM1 was comparable to that observed in ammonium-supplemented Burk’s medium in terms of maximum specific growth rate but differed in final biomass (Fig. 6). In AZM1, biomass increased markedly after the initial growth phase and reached a higher final level. This was accompanied by rapid sucrose consumption and complete ammonium depletion by 36 h, together with transient ethanol accumulation, consistent with respiro-fermentative metabolism followed by ethanol reassimilation. In contrast, growth in ammonium-supplemented Burk’s medium was associated with slower sucrose consumption and incomplete ammonium utilization, with 6.53 ± 1.07 mM ammonium remaining after 48 h (Fig. 6). The two media also exhibited distinct pH profiles: in ammonium-supplemented Burk’s medium, the pH decreased rapidly to 2.65 ± 0.1 within 12 h, whereas in AZM1 it remained at 6.3 ± 0.21 at the same time point, and declined gradually to 3.5 ± 0.24 at the end of cultivation (48 h).

**Fig. 7:**
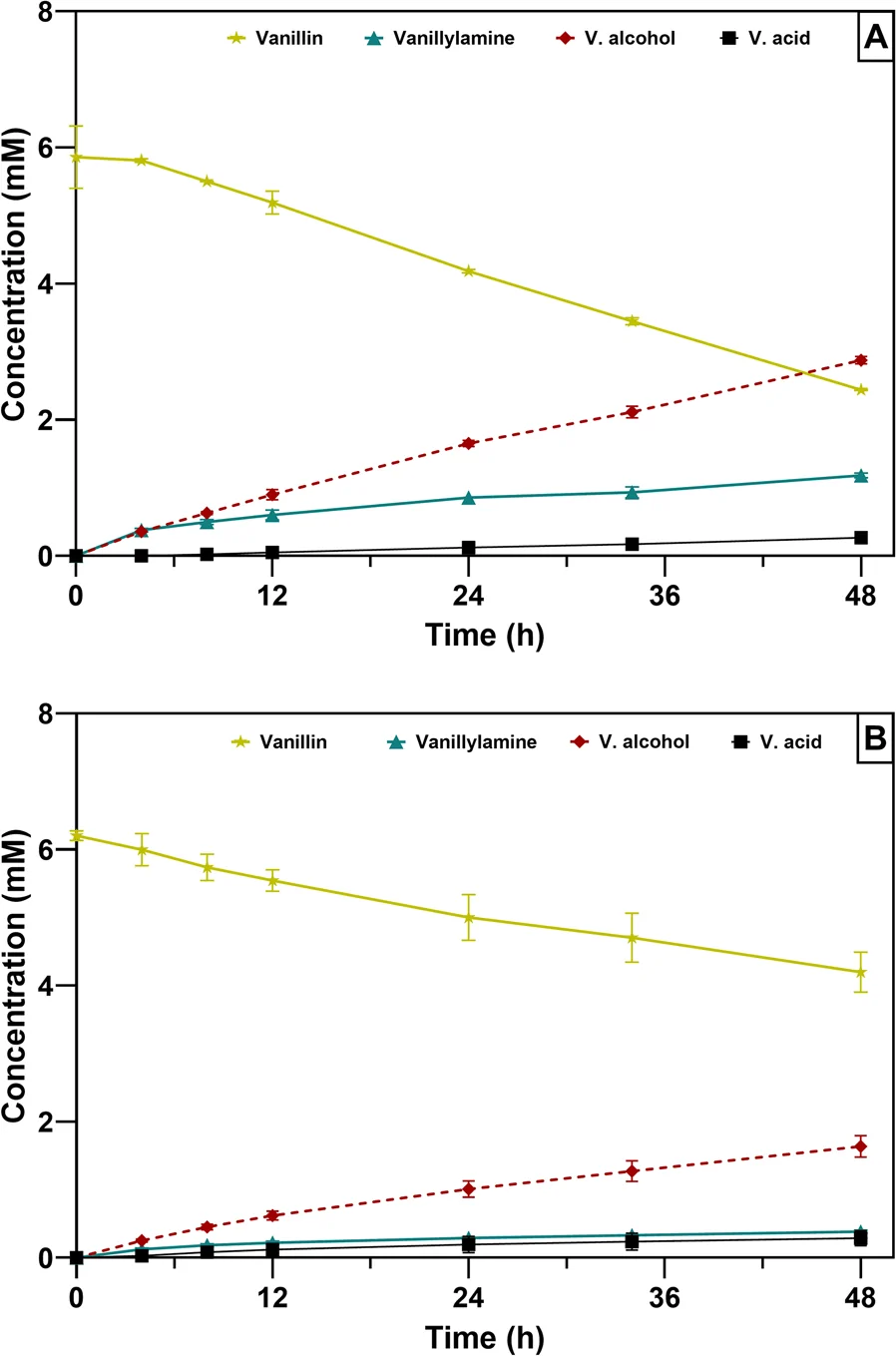
Whole-cell bioconversion of vanillin to vanillylamine by *S. cerevisiae* (TMBNM033 strain) in **A** AZM1 and **B** ammonium-supplemented modified Burk’s medium. Data are presented as mean ± SD of biological replicates (*N* = 2)

The engineered *S. cerevisiae* strain TMBNM033 [9] was subsequently evaluated for whole-cell bioconversion of vanillin to vanillylamine using AZM1-grown cells. In contrast to wild-type yeast, which is unable to produce vanillylamine, this strain expresses amine transaminase from *Chromobacterium violaceum* (Cv-ATA) [24], together with alanine dehydrogenase from *Bacillus subtilis* (Bs-AlaDH) [25]. Furthermore, the strain has a deletion of alcohol dehydrogenase 6 (*ADH6*), which is a major NADPH-dependent vanillin reductase. Ethanol was chosen as co-substrate over sucrose or glucose since it has previously been found to promote amine formation by yeast expressing Cv-ATA [9, 26]. Under semi-aerobic conditions, vanillin was partially converted over 48 h, yielding 0.81 ± 0.12 mM vanillylamine (Table 2), with vanillyl alcohol as the main by-product. Under anaerobic conditions, conversion increased to 58.24 ± 3.43%, and vanillylaine reached 1.18 ± 0.03 mM (Fig. 7; Table 2). Still, a large amount of vanillic alcohol was formed as by-product. In contrast, cultivation and subsequent bioconversion in ammonium-supplemented modified Burk’s medium showed reduced biocatalytic performance, with lower vanillin conversion, lower vanillylamine titres, and increased formation of by-products. This resulted in a reduced product-to-by-product ratio compared to AZM1, indicating that the physiological state established during growth strongly influences subsequent whole-cell bioconversion performance. Vanillic acid formation remained low in all bioconversions, demonstrating low activity of endogenous vanillin dehydrogenase in yeast under the applied conditions.

**Table 2:** Whole-cell bioconversion of vanillin to vanillylamine by TMBNM033 in AZM1 and modified Burk’s media supplemented with ammonium

Conditions	Vanillin conversion (%) *	V. amine titer, 48 h (mM)		V. amine yield (mol/mol)
AZM1, oxygen-limited	46.74 ± 13.30	0.81 ± 0.12		0.33 ± 0.05
AZM1, anaerobic	58.24 ± 3.43	1.18 ± 0.03		0.35 ± 0.06
M-Burk’s, anaerobic	32.34 ± 3.96	0.38 ± 0.02		0.19 ± 0.03

Cultivations were performed for 48 h using 5–6 mM vanillin and 10 g/L ethanol as cosubstrate under anaerobic and oxygen-limited conditions

*Vanillin conversion (%) = (Initial vanillin concentration (0 h) - Residual vanillin concentration (48)) x 100 / Initial vanillin concentration (0 h)

## Discussion

In this study, ammonium generated by *A. vinelandii* was demonstrated to sustain growth of *S. cerevisiae* and support whole-cell biocatalysis. Separating nitrogen fixation from product formation yielded a conditioned medium that enabled yeast-based reductive amination of vanillin. The supernatant from ammonium-excreting *A. vinelandii* cultured in modified Burk’s medium was sufficient without further modification, and no inhibitory effects on yeast performance were observed. However, vanillylamine formation remained limited, consistent with the low ammonium concentrations obtained.

Co-cultivation of *A. vinelandii* and *S. cerevisiae* was possible under diazotrophic conditions, and both organisms remained viable for at least 48 h. However, the system was not compatible with vanillylamine production. In monoculture, *A. vinelandii* rapidly oxidised vanillin to vanillic acid, and this activity is expected to dominate in co-culture. Targeted deletion of vanillin dehydrogenase (*vdh*, Avin_14240) in *A. vinelandii* may provide a route to enable vanillylamine production in co-culture operation by eliminating this competing reaction.

Aerobic batch cultivation of *S. cerevisiae* CEN.PK 113-7d in *A. vinelandii*-conditioned medium (AZM1) was comparable to that in ammonium-supplemented Burk’s medium and resulted in a higher biomass yield. Yeast displayed a metabolite profile consistent with respiro-fermentative growth, characterised by ethanol formation during sugar consumption followed by respiratory assimilation of ethanol. The higher final biomass in AZM1 suggests that additional metabolites in the conditioned medium contribute to growth. Previous studies have shown that *A. vinelandii* releases nitrogen-containing compounds, including amino acids, siderophores, and proteins involved in alginate production [27–29], which may contribute to this effect. Acidification proceeded more slowly in AZM1, which remained near neutral during the initial growth phase, whereas ammonium-supplemented cultures decreased to pH values below 2.6. The basis of this difference cannot be assigned to a specific component from the present data but may reflect differences in nitrogen assimilation, buffering capacity, and/or extracellular metabolites generated during diazotrophic growth of *A. vinelandii*. Medium composition strongly influences pH dynamics in *S. cerevisiae*: ammonium-supported growth can cause rapid acidification, whereas organic acids and medium buffering capacity can modify the pH trajectory [30, 31]. Thus, the delayed acidification in AZM1 likely reflects compositional changes introduced during *A. vinelandii* cultivation rather than ammonium availability alone. From a process perspective, this behaviour could reduce the requirement for external pH control, although this would require validation under controlled cultivation conditions.

The yeast whole-cell biocatalyst strain TMBNM033 showed improved performance when grown in AZM1 for the conversion of vanillin to vanillylamine compared to cells cultivated in synthetic ammonium medium. Both product formation and product-to-by-product ratios were higher. In a previous study using the same strain, approximately 3.6 mM vanillylamine was achieved under substantially higher ammonium concentrations [9], indicating that the lower titres observed here are consistent with limitation in ammonium availability. Formation of vanillyl alcohol indicates that endogenous NADPH-dependent reductases compete with the transaminase for vanillin. Improving selectivity will require further strain engineering, for example through deletion of additional vanillin reductases beyond *ADH6*, as shown previously for vanillin-producing yeast [32].

The population dynamics observed in co-culture further illustrate how nitrogen availability shaped the interaction between the two organisms. Under diazotrophic conditions, both populations remained stable with no indication of overt antagonism. When ammonium was supplied externally, *S. cerevisiae* rapidly dominated while *A. vinelandii* declined, coinciding with strong acidification of the medium. These observations suggest that pH acidification by the yeast influence population stability in correlation with ammonium concentration in the bulk liquid. This is in agreement with previous observation where *A. vinelandii* supported growth of the heterotrophic bacterium *Bacillus subtilis*, whereas excess ammonium suppressed this role and promoted dominance of the non-fixing organism [33].

From a process perspective, the yeast production system is limited by the amount of ammonium that can be supplied by the bacterium. The ammonium levels generated by AZBB664 were sufficient for batch cultivation under the conditions tested but remain low for fed-batch or high-cell-density processes. More generally, ammonium sufficiency depends on the balance between nitrogen supply and carbon-driven demand and is therefore expected to become rate limiting for the growth of yeast at higher sugar concentrations. Non-limiting supply would require higher fixation rates or continuous coupling between ammonium generation and consumption. Strain selection is critical in this context, as wild-type *A. vinelandii* ATCC BAA-1303 did not release detectable extracellular ammonium during cultivation, consistent with previous observations that disruption of *nifL* leads to high-ammonium phenotypes [20, 34]. The reduced biomass yield of AZBB664 compared to the wild type reflects the energetic cost of nitrogen fixation and indicates a trade-off between growth and extracellular nitrogen release, which constrains ammonium availability under process conditions.

Alternative strategies may therefore be required. In engineered *A. vinelandii*, urea can be produced as a terminal metabolic product rather than released as excess ammonium [35]. Unlike ammonium, urea is not a central nitrogen metabolite and does not deregulate biological nitrogen fixation and can be rendered inaccessible to the producing organism through deletion of urease. However, the optimal form of nitrogen may differ between the production strain and the downstream whole-cell biocatalyst. Ammonium is a preferred nitrogen source for *S. cerevisiae* and is directly utilised in the transaminase-alanine dehydrogenase cascade used in the TMBNM033 strain. In contrast, *S. cerevisiae* can also utilise urea through intracellular hydrolysis to ammonium, and its assimilation causes less acidification of the medium [36]. The form of nitrogen produced by *A. vinelandii* therefore represents a system-level design parameter that could influence both upstream production stability and downstream reaction performance.

## Conclusions

Here, we demonstrate that the diazotrophic bacterium *A. vinelandii*, engineered for ammonium excretion, can support growth and amine production by an engineered *S. cerevisiae* catalyst. This is demonstrated using vanillylamine as a model product, establishing atmospheric nitrogen as the sole nitrogen source in a yeast-based production system. Co-cultivation under diazotrophic conditions was feasible and revealed stable coexistence, highlighting the potential for modular system integration, whereas decoupling nitrogen fixation from the amine production phase enabled controlled biocatalysis. Notably, growth of yeast in diazotroph-conditioned medium enhanced process performance compared to synthetic mineral medium, indicating functionality beyond simple ammonium supply and its influence on cellular physiology. Together, these results establish a proof-of-principle for integrating biological nitrogen fixation with microbial cell factories and define a general framework for coupling resource generation with downstream biocatalysis in sustainable production systems.

## Supplementary Information

Below is the link to the electronic supplementary material.

Supplementary Material 1.

## Data Availability

All data supporting the findings of this study are available within the paper and its Supplementary Information.

## References

[1] Vogt PF, Gerulis JJ. Amines, Aromatic. Ullmann’s Encyclopedia Industrial Chem. 2000. 10.1002/14356007.a02_037.

[2] Zhang Y, He YC, Ma C. Efficient synthesis of vanillylamine through bioamination of lignin-derived vanillin by recombinant *E. coli* containing ω-transaminase from Caulobacter sp. D5 in dimethyl sulfoxide-water. Bioresour Technol Elsevier Ltd. 2024. 10.1016/j.biortech.2024.131526. 39321936

[3] Kosem N, Ohsaki Y, Watanabe M, Inada M, Song JT, Ishihara T. Exploring *Azotobacter*: a nitrogen-fixing microorganism as a powerhouse for sustainable and green ammonia synthesis. J Appl Microbiol. 2026;137:lxag083. 10.1093/jambio/lxag083. 41880483

[4] Humphreys J, Lan R, Tao S. Development and recent progress on ammonia synthesis catalysts for haber–bosch process. Adv Energy Sustain Res. 2021. 10.1002/aesr.202000043.

[5] Ghavam S, Vahdati M, Wilson IAG, Styring P. Sustainable Ammonia Production Processes. Front Energy Res Front Media S A. 2021. 10.3389/fenrg.2021.580808.

[6] Smith C, Hill AK, Torrente-Murciano L. Current and future role of Haber–Bosch ammonia in a carbon-free energy landscape. Energy Environ Sci. 2020;13:331–44. 10.1039/c9ee02873k.

[7] Zhao J, Li Y, Yi L, Zhang G, Ye B. Yeast-based whole-cell factory for the ecofriendly synthesis of vanillylamine as a critical step toward capsaicin production. ACS Sustain Chem Eng Am Chem Soc. 2023;11:7683–91. 10.1021/acssuschemeng.2c07561.

[8] Kwiatos N, Ossel S, Mutti FG. Synthesis of amines for active pharmaceutical ingredients using the whole-cell factory *Saccharomyces cerevisae*. ChemBioChem. 2026. 10.1002/cbic.202500898 PMC13157890 42107106

[9] Muratovska N, Carlquist M. Recombinant yeast for production of the pain receptor modulator nonivamide from vanillin. Front Chem Eng Front Media S A. 2022. 10.3389/fceng.2022.1097215.

[10] Barney BM, Lee H-I, Dos Santos PC, Hoffman BM, Dean DR, Seefeldt LC. Breaking the N2 triple bond: insights into the nitrogenase mechanism. Dalton Trans. 2006;35:2277–84. 10.1039/b517633f. 16688314

[11] Burgess BK, Lowe DJ. Mechanism of Molybdenum Nitrogenase. Chem Rev. 1996;96:2983–3012. 10.1021/cr950055x. 11848849

[12] Little R, Martinez-Argudo I, Dixon R. Role of the central region of NifL in conformational switches that regulate nitrogen fixation. Biochem Soc Trans. 2006;34:162–4. 10.1042/BST0340162. 16417511

[13] Bali A, Blanco G, Hill S, Kennedy C. Excretion of ammonium by a nifL mutant of *Azotobacter vinelandii* fixing nitrogen. Appl Environ Microbiol. 1992. https://journals.asm.org/journal/aem 10.1128/aem.58.5.1711-1718.1992 PMC195662 1622243

[14] Plunkett MH, Knutson CM, Barney BM. Key factors affecting ammonium production by an *Azotobacter vinelandii* strain deregulated for biological nitrogen fixation. Microb Cell Fact BioMed Cent Ltd. 2020. 10.1186/s12934-020-01362-9. PMC7238568 32429912

[15] Wang Y, Wang W, Sun W, Cao X, Li C. Promoting nitrogen fixation by threonine synthesis in co-culture of *Azotobacter vinelandii* and *Escherichia coli*. Bioresour Technol. 2026;447:134268. 10.1016/j.biortech.2026.134268. 41734851

[16] Pomraning KR, Deng S, Duong RD, Czajka JJ, Bohutskyi P. Identification of *Yarrowia lipolytica* as a platform for designed consortia that incorporate in situ nitrogen fixation to enable ammonia-free bioconversion. Front Industrial Microbiol Front Media SA. 2024. 10.3389/finmi.2024.1473316.

[17] Wilson PW, Knight SG. Experiments in bacterial physiology. 3rd rev. ed. Minneapolis: Burgess Publishing Company; 1952.

[18] Verduyn C, Postma E, Scheffers WA, Van Dijken JP. Effect of benzoic acid on metabolic fluxes in yeasts: a continuous-culture study on the regulation of respiration and alcoholic fermentation. Yeast Wiley Online Libr. 1992;8:501–17. 10.1002/yea.320080703 1523884

[19] Setubal JC, Dos Santos P, Goldman BS, Ertesvåg H, Espin G, Rubio LM, et al. Genome sequence of *Azotobacter vinelandii*, an obligate aerobe specialized to support diverse anaerobic metabolic processes. J Bacteriol. 2009;191:4534–45. 10.1128/JB.00504-09. 19429624 PMC2704721

[20] Barney BM, Plunkett MH. Rnf1 is the primary electron source to nitrogenase in a high-ammonium-accumulating strain of *Azotobacter vinelandii*. Appl Microbiol Biotechnol Springer Sci Bus Media Deutschland GmbH. 2022;106:5051–61. 10.1007/s00253-022-12059-x. 35804159

[21] Entian K-D, Kötter P. 25 Yeast genetic strain and plasmid collections. In: Stansfield I, Stark MJR, editors. Methods in Microbiology. Academic; 2007. 629–66. 10.1016/S0580-9517(06)36025-4.

[22] Bradford MM. A rapid and sensitive method for the quantitation of microgram quantities of protein utilizing the principle of protein-dye binding. Anal Biochem. 1976;72:248–54. 10.1016/0003-2697(76)90527-3. 942051

[23] George SE, Costenbader CJ, Melton T. Diauxic growth in *Azotobacter vinelandii*. J Bacteriol Am Soc Microbiol. 1985. 10.1128/jb.164.2.866-871.1985. 164:866 – 71. PMC214331 3863813

[24] Kaulmann U, Smithies K, Smith MEB, Hailes HC, Ward JM. Substrate spectrum of ω-transaminase from *Chromobacterium violaceum* DSM30191 and its potential for biocatalysis. Enzyme Microb Technol. 2007;41:628–37. 10.1016/j.enzmictec.2007.05.011.

[25] Koszelewski D, Lavandera I, Clay D, Guebitz GM, Rozzell D, Kroutil W. Formal asymmetric biocatalytic reductive amination. Angewandte Chemie Wiley. 2008;120:9477–80. 10.1002/ange.200803763. 18972473

[26] Hagman A, Stenström O, Carlström G, Akke M, Grey C, Carlquist M. Biocatalytic reductive amination with CRISPR-Cas9 engineered yeast. Sci Rep Nat Res. 2025. 10.1038/s41598-025-01182-0. PMC12081890 40374732

[27] Gimmestad M, Steigedal M, Ertesvåg H, Moreno S, Christensen BE, Espin G, et al. Identification and characterization of an *Azotobacter vinelandii* type I secretion system responsible for export of the AlgE-type mannuronan C-5-epimerases. J Bacteriol. 2006;188:5551–60. 10.1128/JB.00236-06. 16855245 PMC1540039

[28] Revillas JJ, Rodelas B, Pozo C, Martínez-Toledo MV, González López J. Production of amino acids by *Azotobacter vinelandii* and *Azotobacter chroococcum* with phenolic compounds as sole carbon source under diazotrophic and adiazotrophic conditions. Amino Acids. 2005;28:421–5. 10.1007/s00726-004-0153-x. 15731884

[29] Villa JA, Ray EE, Barney BM. *Azotobacter vinelandii* siderophore can provide nitrogen to support the culture of the green algae Neochloris oleoabundans and Scenedesmus sp. BA032. FEMS Microbiol Lett. 2014;351:70–7. 10.1111/1574-6968.12347. 24401035

[30] Prins RC, Billerbeck S. A buffered media system for yeast batch culture growth. BMC Microbiol. 2021;21:127. 10.1186/s12866-021-02191-5. 33892647 PMC8063419

[31] Torija MJ, Beltran G, Novo M, Poblet M, Rozès N, Mas A, et al. Effect of organic acids and nitrogen source on alcoholic fermentation: Study of their buffering capacity. J Agric Food Chem. 2003;51:916–22. 10.1021/jf020094r. 12568549

[32] Xin X, Zhang RK, Liu SC, He ZJ, Liu RY, Lan HN, et al. Engineering yeast to convert lignocellulose into vanillin. Chem Eng J Elsevier B V. 2024. 10.1016/j.cej.2024.149815.

[33] Leroux J, Beauregard PB, Bellenger JP. *Azotobacter vinelandii* N2 fixation increases in co-culture with the PGPR *Bacillus subtilis* in a nitrogen concentration-dependent manner. Appl Environ Microbiol Am Soc Microbiol. 2024. 10.1128/aem.01528-24. PMC11654798 39526803

[34] Barney BM, Dietz BR. Precision control of ammonium release in *Azotobacter vinelandii*. Microb Biotechnol. John Wiley and Sons Ltd; 2024. 10.1111/1751-7915.14523 PMC11256883 39023513

[35] Barney BM, Dietz BR. Enhanced urea production in the diazotroph *Azotobacter vinelandii* as a means of stable nitrogen biofertiliser production. Microb Biotechnol John Wiley Sons Ltd. 2025. 10.1111/1751-7915.70187. PMC12229945 40619687

[36] Yang X, Yang Y, Huang J, Man D, Guo M. Comparisons of urea or ammonium on growth and fermentative metabolism of *Saccharomyces cerevisiae* in ethanol fermentation. World J Microbiol Biotechnol Springer Sci Bus Media B V. 2021. 10.1007/s11274-021-03056-9. 33969436

