# Supplementary material for: Coupling bacterial nitrogen fixation to yeast whole-cell biocatalysis enables amine production using air as the sole nitrogen source
Source: Microb Cell Fact. 2026 Sep 16;25:183. doi: 10.1186/s12934-026-03118-3 (PMC13584442; doi:10.1186/s12934-026-03118-3)
Supplement: Supplementary file 1 [file 12934_2026_3118_MOESM1_ESM.docx]

**Supplementary information**

**Engineering a whole-cell biocatalyst system for sustainable vanillylamine production using *Azotobacter vinelandii* and *Saccharomyces cerevisiae***

Khaled Youssef^1*^, Ed W.J. van Niel^1^, Brett Barney^2,3^ and Magnus Carlquist^1*^

1. Biotechnology and Applied Microbiology, Department of Process and Life Science Engineering, Lund University, P.O. Box 124, Lund SE-221 00, Sweden.
2. Department of Bioproducts and Biosystems Engineering, University of Minnesota, St. Paul, Minnesota, USA.
3. Biotechnology Institute, University of Minnesota, St. Paul, Minnesota, USA.

*** Corresponding authors**: [khaled.youssef@ple.lth.se](mailto:khaled.youssef@ple.lth.se)

[magnus.carlquist@ple.lth.se](mailto:magnus.carlquist@ple.lth.se)

**Figure s1.** Growth of *S. cerevisiae* (CEN.PK113-7D) in modified Burk’s and Delft media under ammonium-replete conditions. Data are presented as means ± SD of biological replicates (N=2).

**Table S1. Detailed chemical composition comparison between Burk's, Delft, and modified Burk's media used in this study.**

| Compound | Burk's (g/l) | Delft (g/l) | Modified Burk's (g/l) |
| --- | --- | --- | --- |
| K_2_HPO_4_ | 0.8 | - | 0.8 |
| KH_2_PO_4_ | 0.2 | 3 | 0.2 |
| CaSO_4_ . 2H_2_O | 0.016 | - | 0.016 |
| FeCl_3_ . 6H_2_O | 0.012 | - | 0.012 |
| Na_2_MoO_4_ . 2H_2_O | 0.003 | - | 0.003 |
| MgSO_4_. 7H_2_O | 0.41 | 0.5 | 0.41 |
| Sucrose / Glucose | 20 | 20 | 20 |
| (NH_4_)_2_SO_4_ | - | 5 | 1 |
| Trace element solution ***** | - | 1% (v/v) | 1% (v/v) |
| Vitamin solution ***** | - | 0.1% (v/v) | 0.1% (v/v) |

***-** The detailed chemical compositions for these stock solutions are described in the 'Strains and media' section of the Materials and Methods.

**Table S2**: Kinetic parameters and statistical grouping (one-way ANOVA and Tukey HSD) of *S. cerevisiae* growth

| Ammonium (mM) | µmax (h^-1^. mean ±SD) | p-value | Tukey group |
| --- | --- | --- | --- |
| 1 | 0.063 ± 0.005 | - | e |
| 1.5 | 0.081 ± 0.013 | 0.755 | de |
| 2 | 0.084 ± 0.016 | 0.99 | de |
| 2.5 | 0.121 ± 0.028 | 0.096 | cd |
| 3 | 0.143 ± 0.019 | 0.561 | bc |
| 5 | 0.182 ± 0.019 | 0.069 | ab |
| 18.78 | 0.186 ± 0.012 | 0.999 | a |

**Table S3. Statistical comparison of diazotrophic growth of *A. vinelandii* ATCC BAA-1303 and AZBB664 over time.**

Least-squares mean OD_600_ values are shown for *A. vinelandii* ATCC BAA-1303 and AZBB664 cultivated in Burk’s medium without added fixed nitrogen at the indicated time points. The table includes the estimated mean difference between strains (ATCC BAA-1303 − AZBB664), the 95% confidence interval for the mean difference, significance level, adjusted P-value, and the number of biological replicates for each strain. Estimates were obtained from a mixed-effects model fitted to repeated-measures growth data, and pairwise comparisons between strains at individual time points were corrected using the Holm–Šídák method. Significance levels are indicated as ns, not significant, and ****, P < 0.0001.

| Time | ATCC LS mean OD | AZBB664 LS mean OD | Mean differences (ATCC BAA-1303 -AZBB664) | Significance | Adjusted P-Value | ATCC BAA-1303 replicate numbers | AZBB664 replicate numbers |
| --- | --- | --- | --- | --- | --- | --- | --- |
| 0 | 0.298 | 0.286 | 0.012 | ns | >0.9999 | 3 | 5 |
| 12 | 5.54 | 5.372 | 0.168 | ns | >0.9999 | 3 | 5 |
| 14 | 7.72 | 7.46 | 0.26 | ns | >0.9999 | 3 | 5 |
| 18 | 15.07 | 7.71 | 7.357 | **** | <0.0001 | 3 | 5 |
| 24 | 12.87 | 6.99 | 5.877 | **** | <0.0001 | 3 | 5 |
| 36 | 12.17 | 6.15 | 6.017 | **** | <0.0001 | 3 | 5 |
| 48 | 11.57 | 6.1 | 5.467 | **** | <0.0001 | 3 | 5 |
| 60 | 11.75 | 5.76 | 5.99 | **** | <0.0001 | 3 | 5 |
| 72 | 11.77 | 5.79 | 5.977 | **** | <0.0001 | 3 | 5 |
